# Correction: Fatal Prion Disease in a Mouse Model of Genetic E200K Creutzfeldt-Jakob Disease

**DOI:** 10.1371/journal.ppat.1006294

**Published:** 2017-05-03

**Authors:** Yael Friedman-Levi, Zeev Meiner, Tamar Canello, Kati Frid, Gabor G. Kovacs, Herbert Budka, Dana Avrahami, Ruth Gabizon

The authors would like to correct Fig 2E and Fig 5, as changes were made to these figures in preparation for publication that were not indicated in the original figures or figure legends. In Fig 2E, the blot had been spliced so that the Tg/ko, Tg/wt, KO, wt, and RML panels were adjacent to each other. A new experiment was run and the corrected Fig 2 comprises all the lanes of the rerun experiment. Arrows mark which lanes were excluded from the original figure.

**Fig 2:**
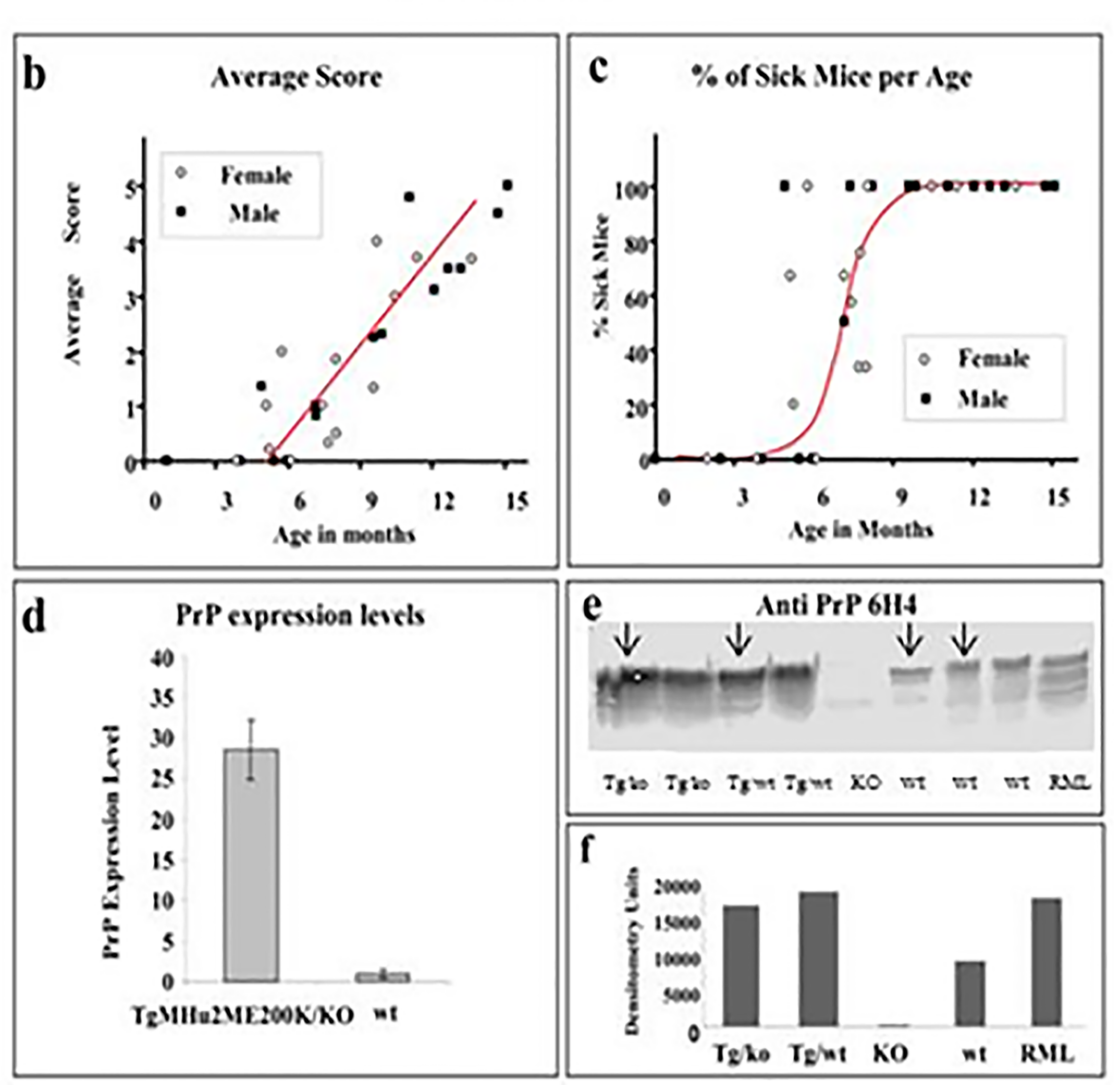
Disease progression in spontaneous disease. **(A)** Average disease onset and death of mice in kinetic studies. **(B)** Aggravation of clinical score of disease as related to the mice's age and gender. Groups of TgMHu2ME199K mice (male and female) were scored for clinical signs from birth to death. Average disease score for each group was plotted against the age elapsed since the mice birth. Closed circles: males, open circles: females. **(C)** Percentage of sick mice in each age group. Groups of mice (as in fig b) in which the average score was at least 1 were plotted against the age of the mice. Closed circles: males, open circles: females. **(D)** Relative PrP mRNA levels, as determined by quantitative RT-PCR for wt and TgMHu2ME199K/ko mice. Each bar represents the average of PrP mRNA normalized against controls genes levels (see methods) in 4 male mice. Statistical bars represent standard error **(E)** Brain homogenates from TgMHu2ME199K/ko, TgMHu2ME199K/wt, PrP ablated, wt C57BL/6 and RML-infected mice were immunoblotted with a PrP 6H4 mAb. **(F)** Relative intensities of the bands as measured by NIH Image J analysis software.

**Fig 5:**
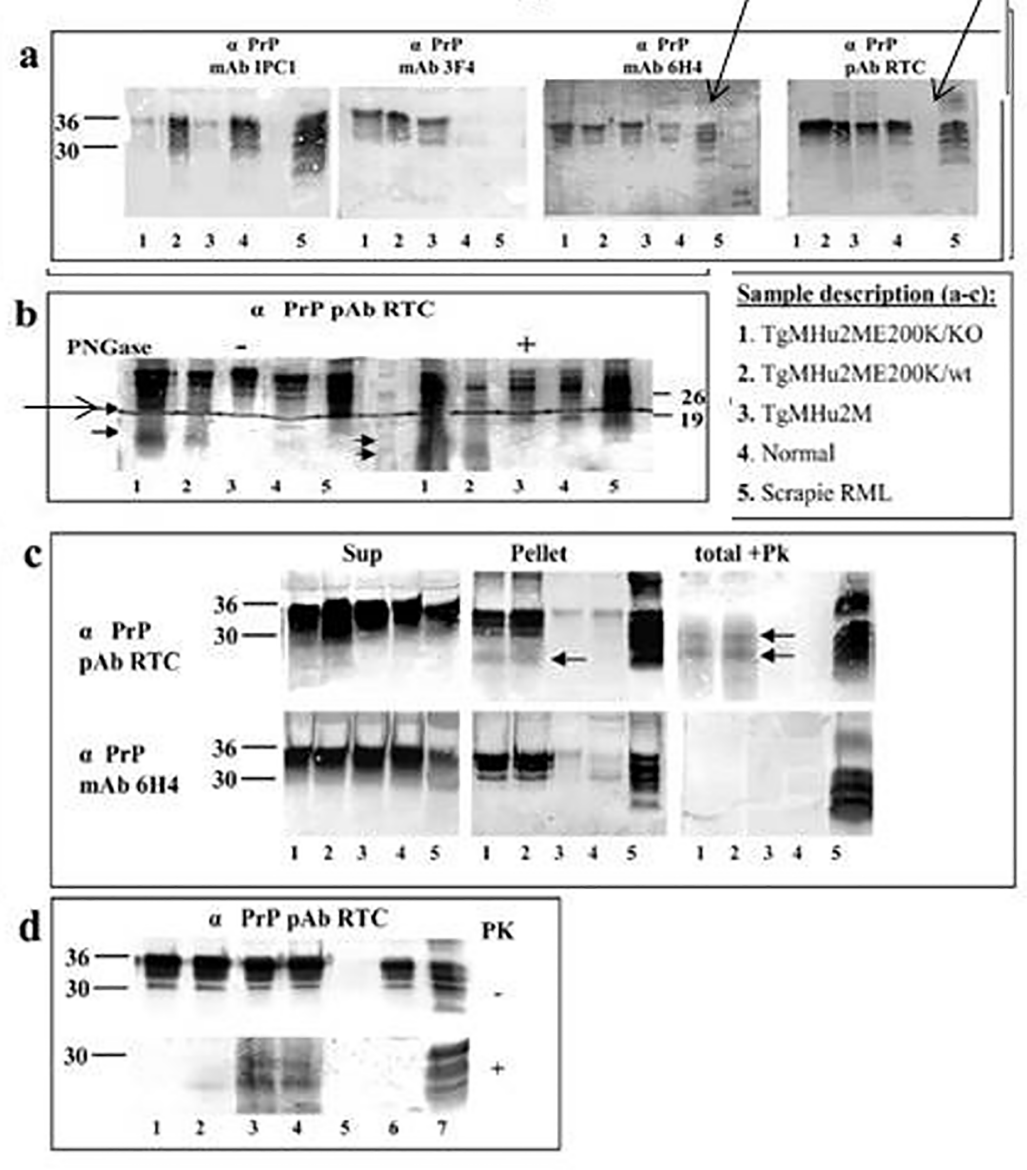
Biochemical Characterization of PrP in TgMHu2ME199K mice. Fig 5. Biochemical Characterization of PrP in TgMHu2ME199K mice. **(A)** To establish the PrP specificity of different samples, brain homogenates from 8 months old mice from 1: TgMHu2ME199K/ko, 2: TgMHu2ME199K/wt, 3: TgMHu2M, 4: wt C57BL/6, and 5: RML infected mice, were immunoblotted with several a PrP antibodies (See Fig 3A for a PrP epitope mapping). Arrows demonstrate truncated PrP forms only present in brains of TgMHu2ME199K mice. **(B)** Samples as in panel a were treated in the presence or absence of PNGase and immunoblotted with designated a PrP antibodies. As above truncated PrP forms are demonstrated only in the TgMHu2ME199K mice demonstrated with arrows. **(C)** Samples as in panel a were extracted with sarkosyl and then centrifuged at 100000 g for 1 h, separated into pellets and supernatants. Otherwise, similar samples (denominated as total) were digested with 30 ug/ml PK for 30 min at 37uC. All samples were then immunoblotted with the designated anti PrP antibodies. Arrows demonstrate PK resistant bands in the TgMHu2ME199K samples. **(D)** Samples from TgMHu2ME199K/ko mice at different ages and controls were digested in the presence or absence of 30 ug/ml PK for 30 min at 37uC and immunoblotted with a PrP pAb RTC. #1: 1 month old; #2: 3 months old; #3: 7 months old; #4: another 7 months old sample, #5 PrP ablated mouse; #6 wt mouse, #7 RML infected mouse.

In Fig 5A, the a Prp/mAB 6h4 and a PrP/pAB RTC blots had lanes removed that were not indicated in the original figure or figure legends. The corrected Fig 5A includes the removed lanes, indicated with arrows. In Fig 5B, the a Prp pAB RTC panel had been adjusted for contrast to erase the dark line running through the image. The corrected Fig 5B is the non-adjusted version of the original image.

The authors confirm that these changes do not alter their findings. The authors have provided raw, uncropped original blots for the corrected figures, Fig 2E, Fig 5A, and Fig 5B. In addition, for full transparency, raw uncropped blots have been provided for other blot figures in the manuscript, including Fig 5C, Fig 6, and Fig 7, as Supporting Information. The authors have not provided additional raw blots for Fig 5D because the authors confirm that the blots included in the original Fig 5D were raw, uncropped blots. The raw blots for Fig 5 are original images, and the raw blots for Fig 6 are a combination of original and alternate images. The raw blots for Fig 7 are original images.

## Supporting information

S1 File Uncropped blots for Figs 2, 5, 6 and 7. (PPTX)
