# Supplementary material for: Correction: Fatal Prion Disease in a Mouse Model of Genetic E200K Creutzfeldt-Jakob Disease
Source: PLoS Pathog. 2017 May 3;13(5):e1006294. doi: 10.1371/journal.ppat.1006294 (PMC5415000; doi:10.1371/journal.ppat.1006294)
Supplement: S1 File — (PPTX) [file ppat.1006294.s001.pptx]

## Slide 1
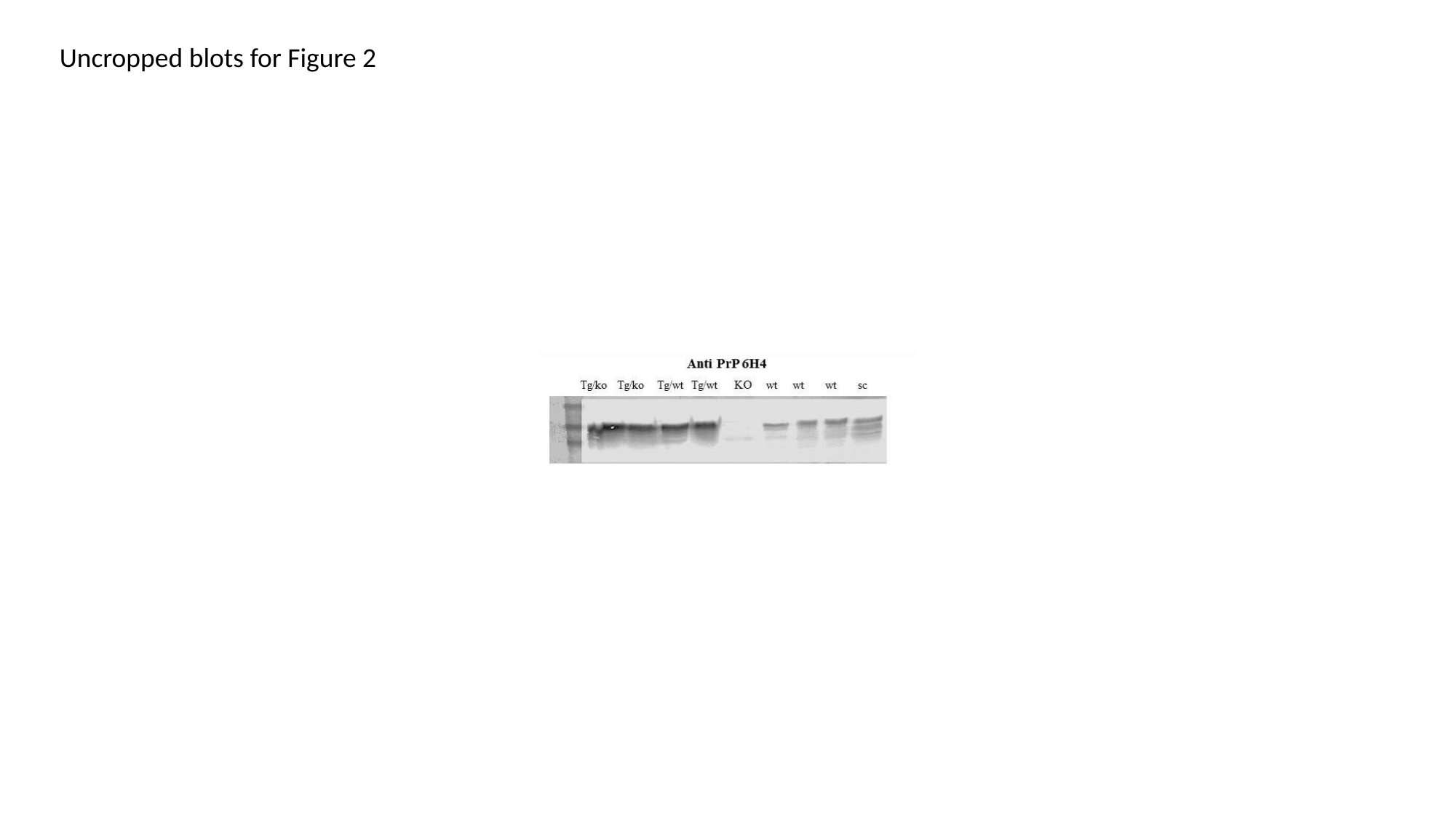

Uncropped blots for Figure 2

## Slide 2
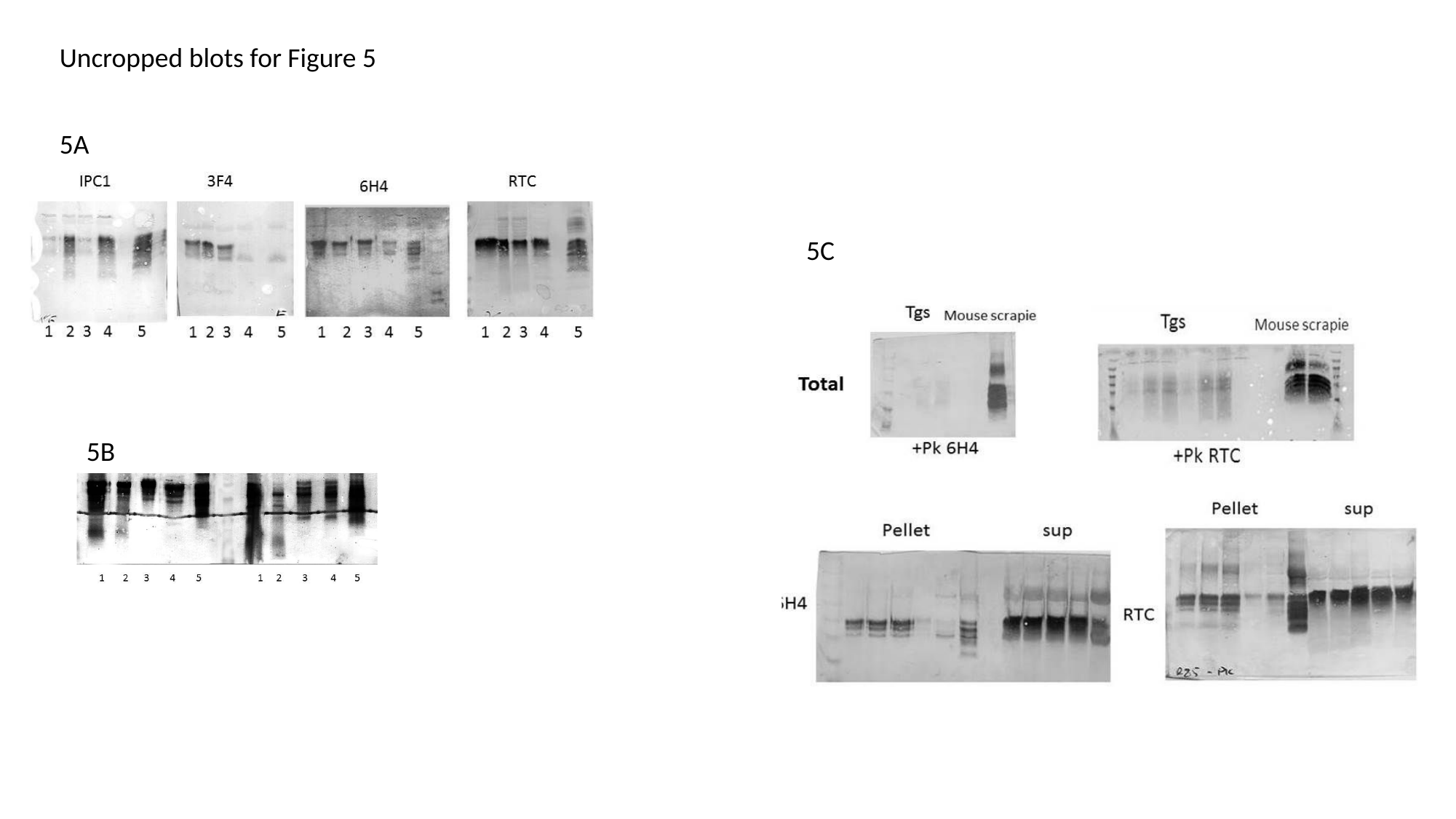

Uncropped blots for Figure 5
5A
5C
5B

## Slide 3
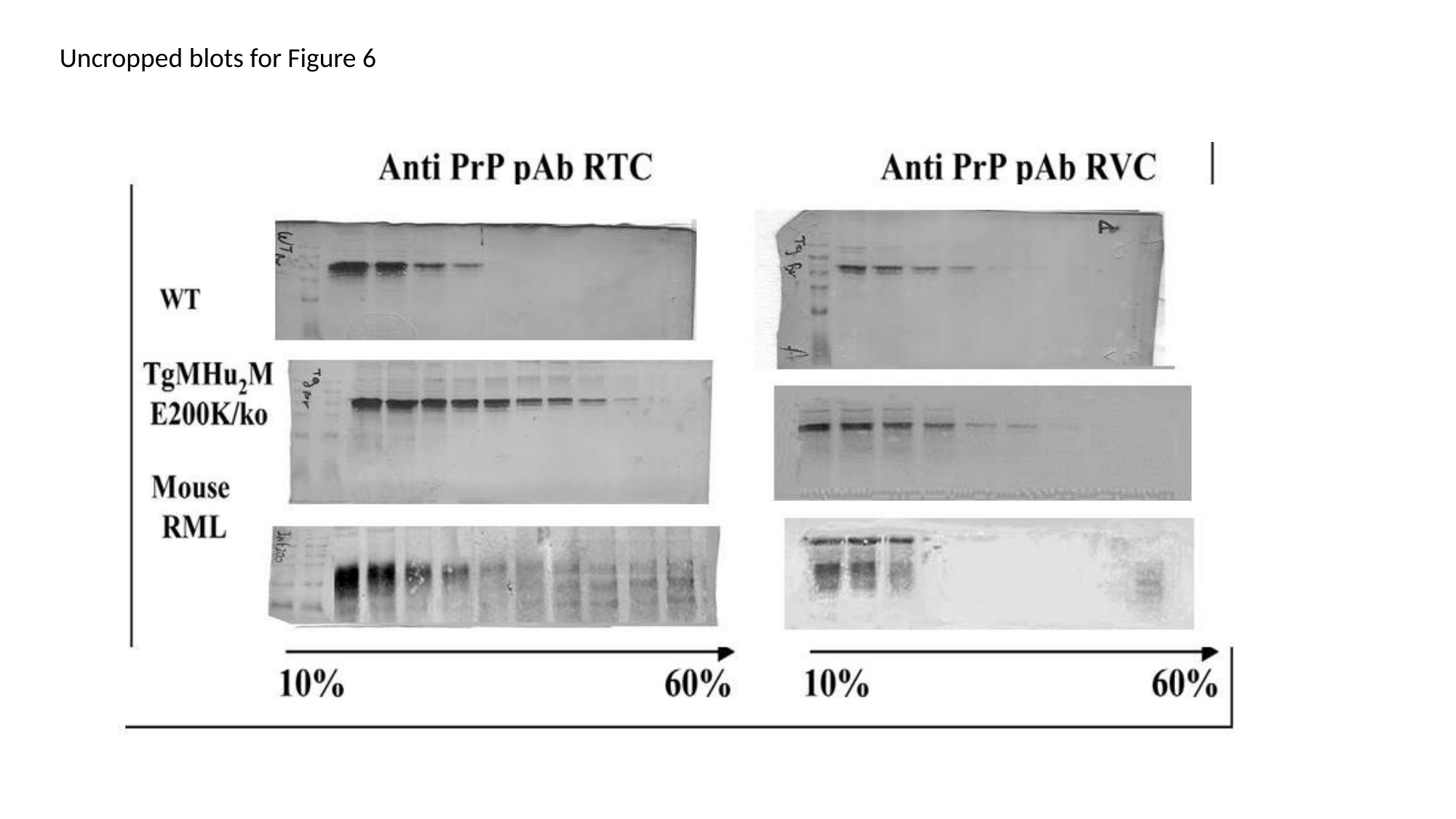

Uncropped blots for Figure 6

## Slide 4
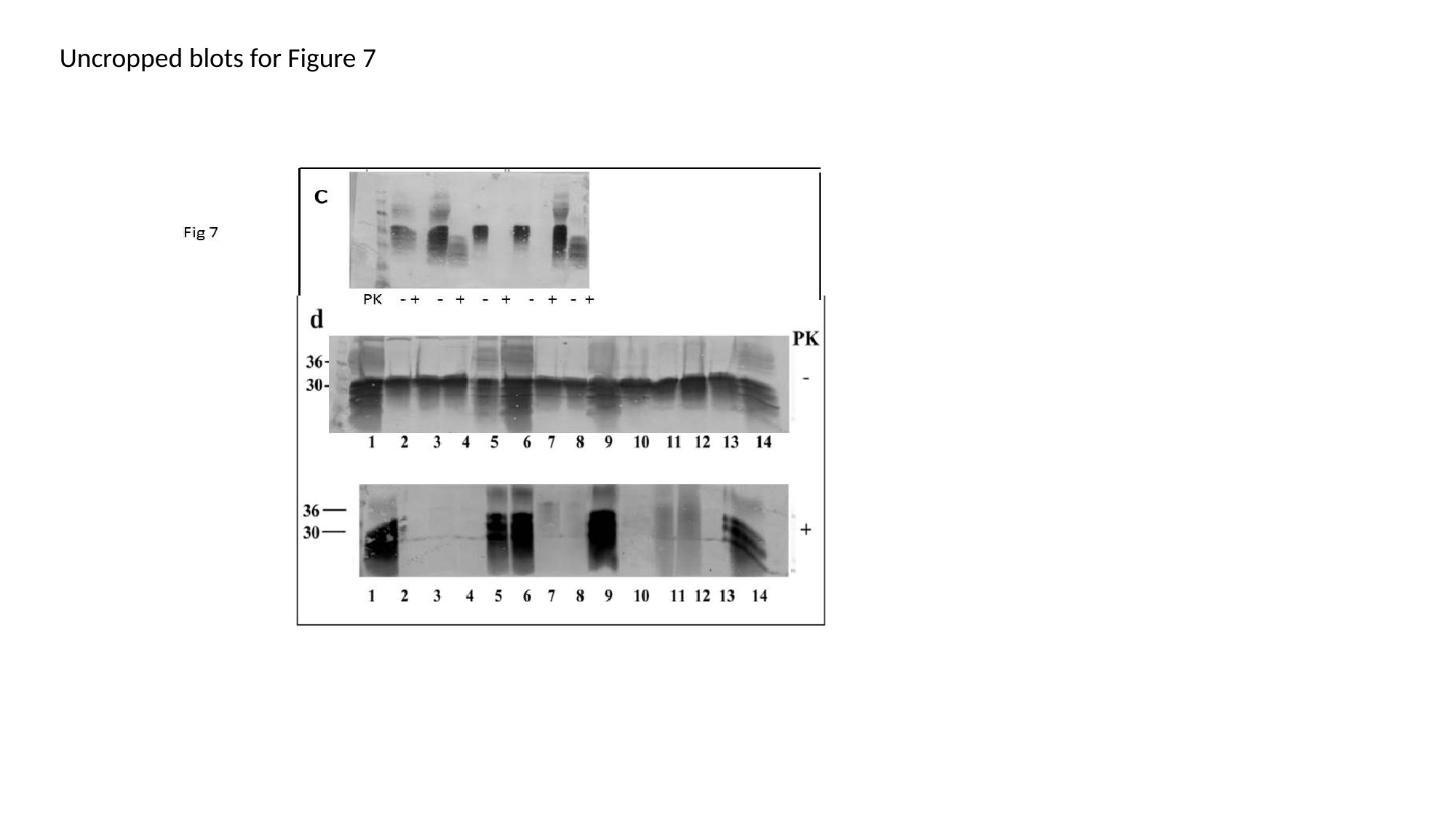

Uncropped blots for Figure 7
